# Research and Remedies

**Published:** 1912-12-07

**Authors:** 


### RESEARCH AND REMEDIES.

### Intussusception.

Some interesting figures about intussusception are given in the *Edinburgh Medical Journal* by Drs. Koch and Oerum, of Copenhagen. It appears that Denmark shares with Great Britain the unenviable distinction of producing more intussusceptions than any other countries, and they have been able to tabulate no fewer than 397 cases. Their conclusions differ in some respects from accepted practice in this country, and are very carefully supported by argument. Thus, for children under one year of age they advocate that non-operative treatment be tried first; abdominal section is reserved for cases which do not respond to this. They describe minutely what they mean by non-operative treatment, and certainly it is a very different method to any that has been practised here. Two-thirds of the cases (in children under a year) can be thus cured, according to these authors. Should any doubt exist as to the successful reduction of the intussusception, then operation is, of course, indicated. Secondary operations are also permissible when the bloodless treatment fails. In dealing with children over one year the risks of operation are found to be so greatly diminished that the Danish surgeons prefer to adopt it at the earliest possible moment. If the case is first seen after the second day then manipulative treatment is allowable, secondary operation being reserved for any cases of failure by the former plan.

### Details of the Manipulative Treatment.

In view of the remarkable statistics which these authors produce concerning the manipulative methods of treatment it is of interest to know what those methods are. They are two in number: taxis, and distension of the bowel with water. For both of these deep general anæsthesia is required, and the bladder and rectum must be evacuated. The tumour formed by the invagination is grasped firmly with both hands through the abdominal wall, and an attempt is made to secure reduction by pressing on the apex, if that can be made out. Even if disinvagination does not follow it is thought that œdema may thus be diminished and the water treatment thereby facilitated. Should taxis fail, the nozzle of an enema syringe is pushed as far up the rectum as possible; the child lies with the pelvis raised. The nates are pressed together around the nozzle, and 500 to 1,000 c.c. of water are injected. A hand on the abdomen judges of the pressure thus generated. The water is kept in as long as possible and is then slowly evacuated. The quantity which escapes is measured; abundance of it bodes well for reposition, especially if mixed with fæces. If the tumour has now disappeared, and if distinct purgation takes place, probably success has been achieved; otherwise the treatment is repeated. Should it still fail open operation must be resorted to. This outline of practice is that which no less an authority than Hirschsprung has adopted, and it may well be worth a trial in this country.

### Neo-Salvarsan.

Whatever views will ultimately be accepted by the general consensus of medical opinion concerning the routine treatment of syphilis by salvarsan, at least it is clear that for severe lesions which are not reacting to mercury Ehrlich's remedy is often extremely useful. It is also clear that salvarsan has drawbacks and dangers which were not fully appreciated at the time of its introduction two years ago. With the object of minimising the latter, and also of improving the anti-syphilitic action of the remedy, Ehrlich has been experimenting with modifications of the original "606." He now states that great advantages attach to a compound to which he gives the name of neo-salvarsan (or "914"). This product has one less amido-group in its constitution than has salvarsan itself. It is soluble in water, forming a neutral solution. Three parts of it (by weight) have an action equivalent to that of two parts of salvarsan, as far as toxicity to human beings is concerned; but spirilla and similar parasitic micro-organisms are actually more affected by the new product. The results clinically are said to be excellent; if it turns out that the numerous fatalities caused by salvarsan can be avoided by this new product, a great step forward will have been made.

### Spontaneous Recovery from Malignant Disease.

Two extraordinary cases of spontaneous recovery from advanced malignant disease were recorded in these columns some time ago, and it was then suggested by our contributor that the blood serum of such exceptional cases might well be worth the attention of cancer investigators. We are not aware that anyone has adopted this suggestion, but we venture to repeat it in view of the very remarkable cases of cancer recorded by Theilhaber in the *Deutsch. Med. Wochenschrift* last summer, an account of which is summarised in the *Medical Review*. Four cases of uterine cancer and one case of breast cancer are placed on record, all of them duly verified from a diagnostic standpoint and all of them apparently hopeless clinically. The breast case is, perhaps, an exception to this statement, inasmuch as a radical operation was performed and there was no proof that the glandular lump subsequently formed was really due to malignant metastasis; it might well have been of septic origin, and the cure of the cancer would then have been due to the operation rather than to the *vis medicatrix naturæ*. But in the uterine cases these criticisms hardly apply, and it must be admitted that spontaneous cure of apparently incurable cancers took place. There is nothing improbable in this, as anyone familiar with cancer literature will admit; indeed, none of these four cases is as remarkable as the two cases to which we drew attention in 1909. Since then, it cannot be denied that spontaneous cure of cancer does very rarely occur, it follows surely that research along the lines of its mode of action might be worth while, on the bare chance of its leading to some tangible result.

